# Multi-Pilot Channel Estimation for Orthogonal Time–Frequency Space Systems Based on Constant-Amplitude Zero-Autocorrelation Sequences

**DOI:** 10.3390/s24237588

**Published:** 2024-11-27

**Authors:** Renjie Ju, Yangyanhao Guo, Xiaojuan Hou, Jian He, Ting Li, Zhiqiang Lan, Xiujian Chou

**Affiliations:** 1 Science and Technology on Electronic Test and Measurement Laboratory, School of Instrument and Electronics, North University of China, Taiyuan 030051, China; sz202206019@st.nuc.edu.cn (R.J.); s202206044@st.nuc.edu.cn (Y.G.); drhejian@nuc.edu.cn (J.H.); chouxiujian@nuc.edu.cn (X.C.); 2 School of Communication and Information Engineering, Nanjing University of Posts and Telecommunications, Nanjing 210003, China; lit@njupt.edu.cn; 3 School of Future Science and Engineering, Soochow University, Suzhou 215299, China

**Keywords:** OTFS, delayed Doppler domain, channel estimation, CAZAC

## Abstract

Future communication systems must support high-speed mobile scenarios, while the mainstream Orthogonal Frequency Division Multiplexing (OFDM) technology faces severe inter-carrier interference in such environments. Therefore, the adoption of Orthogonal Time–Frequency Space (OTFS) modulation in 6G systems is an effective solution. The widely used single-pilot channel estimation in OTFS systems is susceptible to path loss and inaccurate fading coefficient estimation, leading to reduced estimation accuracy, signal distortion, and degraded overall system communication quality. To address this problem, this paper proposes a Constant-Amplitude Zero-Autocorrelation (CAZAC) sequence-based multi-pilot OTFS channel estimation scheme. The proposed method inserts multiple low-power pilots in the delayed Doppler domain (DD) and employs joint signal processing at the receiver to effectively suppress noise, thereby significantly improving the accuracy and reliability of channel estimation. Additionally, this paper analyzes the impact of CAZAC sequence length on estimation performance and provides reasonable parameter selection recommendations. In summary, this work proposes an innovative solution to the channel estimation challenge in OTFS systems, laying a solid theoretical foundation for the realization of future high-speed mobile communication technologies such as 6G, with important academic value and application prospects.

## 1. Introduction

With the rapid development of social informatization and digitalization, future communication systems must support various high-speed application scenarios, such as high-definition video transmission and virtual reality. However, the orthogonal frequency division multiplexing (OFDM) technique [1,2,3,4], which is widely used in 4G and 5G mobile communication systems, suffers from serious intra-carrier interference problems in high-mobility environments [5,6]. To address the limitations of OFDM in high-mobility scenarios, the orthogonal time–frequency space (OTFS) technique is proposed. OTFS converts a time-varying fading channel into a non-fading and time-independent channel by modulating the information in the delayed Doppler (DD) domain, showing significant countermeasures against the Doppler effect and frequency-selective fading in high-speed mobile scenarios [7,8,9].

The core of the OTFS system model lies in the use of transformations to convert the time-varying fading channel to the DD domain, such that all symbols in the transmission unit experience almost the same and slowly varying sparse channel [10]. In addition, the peak-to-average ratio (PAPR) of OTFS signals is lower compared to OFDM due to the uniform expansion of all modulated symbols in the time–frequency domain [11]. However, the channel estimation and frequency guide design of OTFS systems are more challenging and have higher algorithmic complexity than OFDM techniques [12]. There is still room for further improvement in the performance of current channel estimation schemes, especially when constrained by non-ideal conditions, including the case of fractional Doppler in the DD domain [13].

Accurate channel estimation is essential to achieve high-performance OTFS transmission. To address this need, Raviteja et al. [14] inserted a single guide frequency and guard interval in the delayed Doppler domain to handle the maximum Doppler shift and maximum delay extension. Zhao et al. [15] derived the feasibility of threshold-assisted channel estimation in OTFS systems and used multi-pulse extraction to achieve diversity gain. These methods are simple and practical, but the spectral efficiency is relatively low. In order to improve the spectral efficiency, Yuan and Mishra [16,17] abandoned the guard interval and proposed an overlapping guide frequency and data symbol scheme, as well as an iterative channel estimation algorithm with data detection and interference cancellation to mitigate the interference between the data and guide frequencies. However, these single-guide-frequency channel estimation methods still suffer from low estimation accuracy, low spectrum utilization, and weak interference immunity [17].

To solve these problems, Liu et al. [18] proposed an OTFS channel estimation method based on multiple-scattering guide frequencies. The method utilizes the natural scattering characteristics of the signal in the time–frequency domain and obtains channel information by setting multiple-scattering guide-frequency symbols. Although the method does not require additional signal processing overheads, the weak guide-frequency signals are susceptible to noise interference, which affects the estimation accuracy [19].

Currently, the challenges of channel estimation in OTFS systems, such as fractional Doppler dispersion and delay-domain phase differences, lead to the limited performance of traditional single-guide-frequency methods in practical applications [20]. In addition, although the channel estimation method based on embedded frequency guidance reduces the frequency guidance overhead, the high dependence on the frequency guidance signal at low signal-to-noise ratios leads to insufficient estimation accuracy [7].

Therefore, in order to further improve the accuracy and reliability of OTFS channel estimation, this paper proposes a multi-conductor OTFS channel estimation scheme based on CAZAC sequences on the basis of the multi-scattered guide-frequency OTFS channel estimation method. The proposed method uses multiple low-power guided frequencies in the time–frequency delayed Doppler (DD) domain and jointly processes the received guided-frequency signals to estimate the channel parameters. Compared with the existing multi-scattered guided-frequency methods, this method can achieve stronger signal power without increasing the time–frequency resources, which significantly improves the accuracy and reliability of channel estimation [19,20,21,22]. In addition, this method is also applicable to multi-antenna MIMO-OTFS systems, which can further improve the performance [23].

Compared with the multiple-scattering pilot method, the proposed multi-pilot OTFS channel estimation method based on CAZAC sequences has the following advantages: (1) stronger signal power can be obtained, which improves the estimation accuracy and reduces the normalized mean square error by 20%; (2) there is no need for additional signal processing overheads, and thus the spectral efficiency is high, with an improvement of about 30% in spectral efficiency; and (3) in the future, it can be applied to multi-antenna MIMO-OTFS systems, which is more practical. These advantages make the proposed method ideal for high-precision channel estimation in OTFS systems.

## 2. OTFS System Model

**Figure 1:**
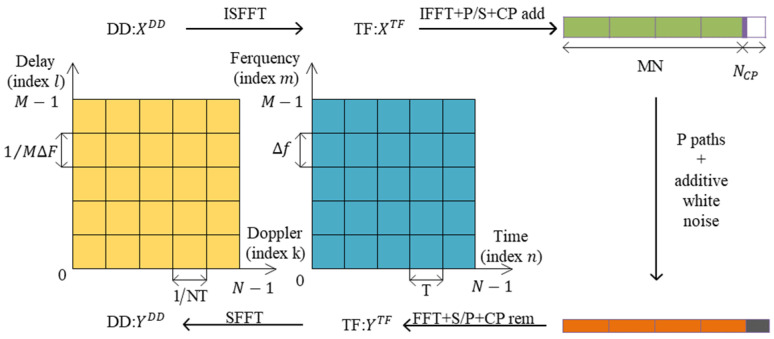
OTFS system model.

The flow of the OTFS system is shown in Figure 1. In Figure 1, SFFT stands for Symplectic Finite Fourier Transform, which is used to transform the signals in the time–frequency domain (TF domain) back to the time–delay Doppler domain (DD domain). ISFFT stands for Inverse Symplectic Finite Fourier Transform, which converts the data in the DD domain to the symbols in the TF domain. IFFT stands for Inverse Fast Fourier Transform, which is used to convert the signals in the frequency domain back to the time domain. To describe the entire process of OTFS modulation and demodulation concisely, the delay Doppler domain input–output relationship and the specific expression of the equivalent channel matrix are obtained. This part of the formula is based on matrix representation and discrete time.

In OTFS modulation, the information symbols (i.e., QAM, $A=a_{1},a_{2},\ldots ,a_{Q}$) are first directly mapped to the delay Doppler (DD) domain, forming the data matrix $X^{DD}\in C^{M\times N}$. This data matrix X is then mapped to the time–frequency domain, generating the time–frequency domain signal matrix $X^{TF}\in C^{M\times N}$. Next, OFDM modulation is performed, transforming the time–frequency domain signal matrix $X^{TF}$ to the time domain signal matrix $S=G_{tx}F_{M}^{H}X^{TF}=G_{tx}F^{DD}F_{N}^{H}$, where $G_{tx}$ is the transmitter pulse waveform and $F^{H}$ is the inverse Fourier transform matrix. Finally, the time domain signal matrix s is expanded by column priority to form the final transmitted signal vector s. This complete transmitter-side processing flow of the OTFS system provides a solid foundation for further research on OTFS system performance analysis and channel estimation algorithms.

(1) $$s=vec(S)=(F_{N}^{H}⊗G_{tx})x^{DD}$$

where $x^{DD}=vec(X^{DD})$, and the symbol ⊗ represents the circular convolution operation between two functions. A cyclic prefix is added to the time domain signal s before transmission, and the cyclic prefix length is determined based on the maximum multipath delay of the channel. The transmitted data signal passes through a bidirectional selective fading channel, and the loop prefix is removed at the receiving end to obtain the received signal vector r.

In the discrete time domain, the expression is as follows:

(2) $$r(n)=\sum_{i=1}^{P}h_{i}e^{j2\pi \frac{k_{i}(n-l_{i})}{MN}}s({\left[n-l_{i}\right]}_{MN})+w(n)$$

where P represents the number of paths, and $h_{i}$, $l_{i}$, and $k_{i}$ represent the channel gain of the i path, the delay of the integer, and the Doppler shift of the integer, respectively; the above equation is written as a vector as follows:

(3) r=Hs+w

where $H=\sum_{i=1}^{P}h_{i}\Pi^{l_{i}}\Delta^{k_{i}}\in C^{MN\times MN}$.

(4) $$\Pi ={\left[\begin{array}{cccc} 0 & \cdots & 0 & 1\\ 1 & \ddots & 0 & 0\\ \vdots & \ddots & \ddots & \vdots \\ 0 & \cdots & 1 & 0 \end{array}\right]}_{MN\times MN}$$

(5) $$\Delta =diag[z^{0},z^{1},\ldots z^{MN-1}]$$

where $z=e^{\frac{j2\pi }{MN}}$.

Then, the reverse operation corresponding to the transmitter is performed, the received signal is serialized and transformed into $R=vec^{-1}(r)$, and then the signal is converted to the time–frequency domain and DD domain in turn, as shown below:

(6) $$Y^{TF}=F_{M}G_{rx}R$$

(7) $$Y^{DD}=F_{M}^{H}Y^{TF}F_{N}=G_{rx}RF_{N}$$

where $G_{rx}$ corresponds to matching filtering. To reflect the effect of the channel, the received signal matrix $Y^{DD}$ is written in vectorized form:

(8) $$y^{DD}=vec(Y^{DD})=(F_{N}⊗G_{rx})r=H_{eff}x^{DD}+\overset{⌢}{w}$$

where $H_{eff}$ and $\overset{⌢}{w}$ represent the effective channel and noise vector, respectively. When using a rectangular wave, the channels are represented as follows:

(9) $$H_{eff}=(F_{N}⊗I_{M})H(F_{N}^{H}⊗I_{M})=\sum_{i=1}^{P}h_{i}T^{i}$$

where $T^{i}(F_{N}⊗I_{M})\Pi^{l_{i}}\Delta^{k_{i}}(F_{N}^{H}⊗I_{M})$. According to the expression available in the literature [13], the result is related to the delay and Doppler shift of each path. According to Heff, a two-dimensional convolution relationship between the input and output of the DD domain can be obtained:

(10) $$Y^{DD}(l,k)=\sum_{i=1}^{P}h_{i}\alpha_{i}(l,k)X^{DD}({\left[l-l_{i}\right]}_{M},{\left[k-k_{i}\right]}_{N})+v(l,k)$$

where v(l,k) represents delay Doppler domain noise and $\alpha_{i}(l,k)$ is represented as follows:

(11) $$\alpha_{i}(l,k)=\{\begin{array}{c} e^{-j2\pi \frac{k}{N}}z^{k_{i}({\left[l-l_{i}\right]}_{M})},l<l_{i}\\ z^{k_{i}({\left[l-l_{i}\right]}_{M})},l\geq l_{i} \end{array}$$

It can be seen from the formula that the input and output of the OTFS system in the DD domain present a two-dimensional convolutional relationship; the OTFS system shows orthogonal and separable characteristics between channels with a small delay and Doppler resolution, and the receiving end can separate the received signals from different paths to finally obtain the time–frequency domain’s full diversity. From the formula, it can be found that the channel state information is time-independent; that is, the delay Doppler domain channel state information does not change with time [21].

## 3. Motivation and Method

In OTFS systems, single-pilot channel estimation suffers from path loss and severe fading coefficient estimation errors under poor channel conditions [22]. To address this issue, this paper proposes a CAZAC sequence-based multi-pilot OTFS channel estimation method. The proposed approach inserts CAZAC sequence pilots along the Doppler domain, leveraging the characteristics of CAZAC sequences to reduce the complexity of correlation operations. Concurrently, scanning and denoising techniques are applied at the receiver to effectively suppress secondary peak interference, thereby improving the estimation accuracy. Compared to the single-pilot scheme, the multi-pilot approach offers greater flexibility, allowing the number of pilots to be adjusted as per the requirements without being constrained by the peak-to-average power ratio. Furthermore, the proposed scheme can be extended to MIMO-OTFS systems, where orthogonal CAZAC sequences are employed to realize inter-antenna channel estimation, thereby further enhancing the system’s performance and spectral efficiency.

### 3.1. Limitations of Single-Pilot Channel Estimation

**Figure 2:**
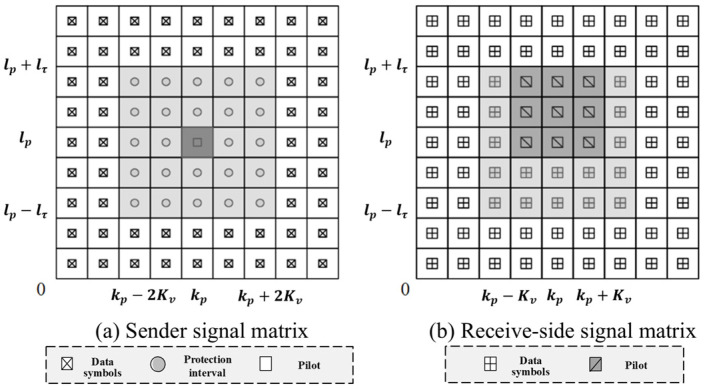
A schematic diagram of the signal matrix at the transceiver end of a single pilot. (**a**) The frame structure of the transmitter containing the pilot, protection interval, and data symbols, and (**b**) the data symbols received at the receiver after passing through the channel.

Based on the literature [14], a pilot symbol $x_{p}$ and zero values $N_{n}$ are placed in each OTFS transmission frame as protection symbols and the remaining positions $MN-N_{n}-1$ are used to insert the transmitted data symbols, as shown in Figure 2.

The symbol arrangement shown in Figure 2a makes it possible to separate two different groups of receiver symbols at the receiver: the first group contains the pilot and protection symbols for channel estimation as shown in Figure 2b in the region containing the pilot, and the remaining part contains the transmitted data symbols for data detection. Specifically, by inserting the pilot and protection interval in the DD domain, the transmitted data take the form of the following equation:

(12) XDD[l,k]={xp,k=kp,l=lp0,kp−2kv≤k≤kp+2kv lp−lτ≤l≤lp+lτxd[l,k],otherwise

where $x_{p}$ indicates that the pilot can be placed at any grid point in the delayed Doppler grid, $0\leq l_{p}\leq M-1$, and $0\leq k_{p}\leq N-1$. For ease of presentation, $0\leq k_{p}-2k_{v}\leq k_{p}$$\leq k_{p}+2k_{v}\leq N-1$ and $0\leq l_{p}-l_{r}\leq l_{p}\leq l_{p}+l_{r}\leq M-1$ are specified, as shown in Figure 2a, with a value of 0 placed at the guard interval and d denoting the transmitted data. t and v denote the integer maximum channel delay and Doppler shift, respectively, and the insertion of the guard interval allows the pilot and data to pass through the channel without overlapping, improving channel estimation accuracy. In this case, the protection interval has $N_{n}=(2l_{\tau }+1)(4k_{v}+1)-1$ zero values.

According to the equation, it can be derived that the pilot is transmitted through the channel and the received signal form in the range $k\in [k_{p}-k_{v},k_{p}+k_{v}],l\in [l_{p},l_{p}+l_{\tau }]$ is as follows:

(13) $$Y^{\mathrm{DD}}[l,k]=\sum_{i=1}^{P}h_{i}x_{p}\delta \left(l-l_{p}-l_{i}\right)\delta \left(k-k_{p}-k_{i}\right)+v(l,k)$$

If the delay index of path i is $l-l_{p}$ and the Doppler index is $k-k_{p}$, then $\delta (l-l_{p}-l_{i})=1$ and $\delta (k-k_{p}-k_{i})=1$, i.e., $Y^{DD}\left[l,k\right]=h_{i}$; otherwise $Y^{DD}\left[l,k\right]=v\left(l,k\right)$.

Based on the above analysis, single-pilot channel estimation is scanned for the range $k\in [k_{p}-k_{v},k_{p}+k_{v}],l\in [l_{p},l_{p}+l_{\tau }]$.

(14) $$\hat{h}[l,k]=\{\begin{array}{cc} Y^{DD}[l,k]/x_{p}, & \left|Y^{DD}[l,k]\right|\geq \alpha \\ \;0, & \mathrm{otherwise}\; \end{array}$$

where α denotes the positive judgment threshold. Single-pilot channel estimation in channels with severe white noise or fading may result in path misses and excessive errors in fading coefficient estimation.

In the case of a low signal-to-noise ratio, a larger amplitude of the pilot is required to obtain a higher channel estimation accuracy. This problem can be alleviated by increasing the energy of the single pilot, but the energy of the single pilot is limited due to the power peak-to-average ratio limitation. The energy of the pilot in the literature [24] is set to 30 dB, 35 dB, and 40 dB, and the amplitude value of $x_{p}$ cannot be set larger in practical applications. In this paper, we propose multiple-channel estimation to solve this problem. When single-pilot channel estimation is applied to an MIMO-OTFS system [25], it is assumed that there are $N_{t}$ antennas at the transmitter, and the pilot block size is proportional to $N_{t}l_{\tau }k_{v}$ for each antenna transmitting data to the pilot without interference. With a large number of antennas at the base station, the pilot overhead will be huge [14].

### 3.2. Multi-Pilot Channel Estimation Based on CAZAC Sequences

The main idea of single-pilot channel estimation is to search for peaks larger than the threshold that occur in a specific range and estimate the channel state information based on the peaks. The synchronization operation is often carried out in 4G and 5G using some sequences with good correlation properties to generate peaks. In this section, we propose the use of CAZAC sequences to generate peaks to complete the channel estimation.

CAZAC sequences are constant-amplitude zero-autocorrelation sequences. Widely used in OFDM systems, the common ones are the Zadoff–Chu sequence (ZC), Frank sequence, Chirp sequence, and Golomb sequence. In this paper, ZC sequences are used as follows:

(15) $$C(k)=\{\begin{array}{cc} e^{\frac{j\pi rk^{2}}{N_{1}}} & N_{1}\%2=0\\ e^{\frac{j\pi rk(k+1)}{N_{1}}} & N_{1}\%2=1 \end{array}$$

where $N_{1}$ is the ZC sequence length, r is reciprocal to $N_{1}$, and in this paper, we choose $N_{1}$ as an even number and r=1. The ZC sequence autocorrelation properties are shown below:

(16) $$\sum_{k=0}^{N_{1}}C(k)C^{*}\left({\left[k+\tau \right]}_{N_{1}}\right)=\{\begin{array}{cc} N_{1} & \tau =0\\ 0 & \tau \neq 0 \end{array}$$

**Figure 3:**
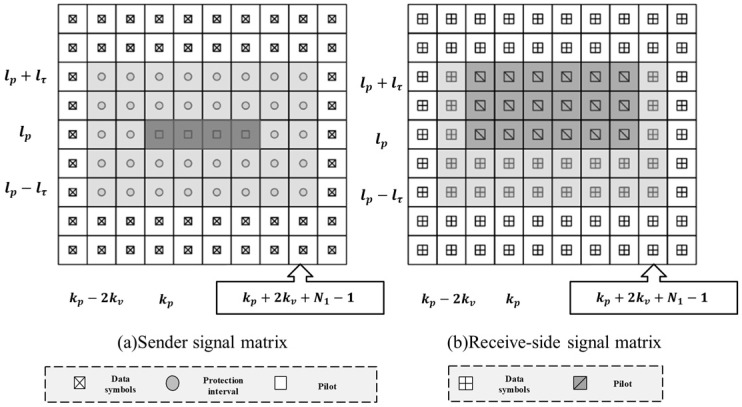
The multi-conductor transceiver signal matrix diagram. (**a**) The frame structure of the transmitter containing the pilot, protection interval, and data symbols, and (**b**) the data symbols received at the receiver after passing through the channel.

Inserting pilot-in and protection intervals in the DD domain in the range $k_{p}-2k_{v}\leq k\leq k_{p}+2k_{v}+N_{1}-1$, $l_{p}-l_{\tau }\leq l\leq l_{p}+l_{\tau }$ and data symbols in the remaining positions gives the following:

(17) $$X^{\mathrm{DD}}[l,k]=\{\begin{array}{cc} e^{\frac{j\pi {\left(k-k_{p}\right)}^{2}}{N_{1}}}, & l=l_{p},k_{p}\leq k\leq k_{p}+N_{1}-1\\ \;\;0, & \mathrm{otherwise}\;\; \end{array}$$

where point $\left(l_{p},k_{p}\right)$ indicates the starting point of the pilot sequence and $2k_{v}\leq k_{p}\leq N-1-2k_{v}-N_{1}$, $l_{\tau }\leq l_{p}\leq M-1-l_{\tau }$, as shown in Figure 3. In this case, the protection interval has a total of $N_{n}=(2l_{\tau }+1)\left(4k_{v}+N_{1}\right)-N_{1}$ zero values.

The CAZAC sequence in Equation (15) is inserted along the direction of the Doppler dimension. It can be found from Equation (10) that when $l=l_{i}$, $a_{i}\left(l,k\right)=1$, i.e., the ZC sequence of the ith path along the Doppler shift direction is transmitted through the channel without phase distortion. At this point, the complexity of correlation operation detection can be reduced. At the receiver side, the received data in the range $k\in [k_{p}-k_{v},k_{p}+k_{v}+N_{1}-1],\;l\in [l_{p},l_{p}+l_{\tau }]$ are scanned for noise removal before channel estimation, as shown below:

(18) $$Y^{\mathrm{DD}}[l,k]=\{\begin{array}{cc} Y^{\mathrm{DD}}[l,k], & |y[k,l]|\geq \beta \\ 0, & \mathrm{otherwise} \end{array}$$

where β is the judgment threshold.

First, we determine the range of data to be scanned. For the Doppler shift index k, the range is $k\in [k_{p}-k_{v},k_{p}+k_{v}+N_{1}-1],l\in [l_{p},l_{p}+l_{\tau }]$, where $k_{p}$ is the center Doppler shift index, $k_{v}$ is the extended range of the Doppler shift, and $N_{1}$ is the number of samples in the delay dimension. For the time delay index l, the range is $\left[l_{p},l_{p}+l_{\tau }\right]$, where $l_{p}$ is the center delay index and $l_{\tau }$ is the extended range of the time delay. And then, a judgment threshold β is set based on the statistical properties of noise to distinguish between signal and noise. Finally, the received data $Y^{\mathrm{DD}}[l,k]$ within the above range are scanned. For each data point y[k,l], if its amplitude |y[k,l]| is greater than or equal to the threshold β, the data point is considered a valid signal and its value is retained; otherwise, the data point is considered noise and its value is set to zero.

This operation has two advantages. One is that the channels are sparse in the DD domain, so only a small number of valid channels do not need to correlate all points in the range $k\in [k_{p}-k_{v},k_{p}+k_{v}+N_{1}-1],l\in [l_{p},l_{p}+l_{\tau }]$. After scanning to reduce the amount of computation, setting a value below the threshold to zero can reduce the number of $N_{1}$ complex multiplications. Secondly, according to the formula, two complete ZC sequences (i.e., both of length $N_{1}$) are correlated to show good intercorrelation properties.

**Figure 4:**
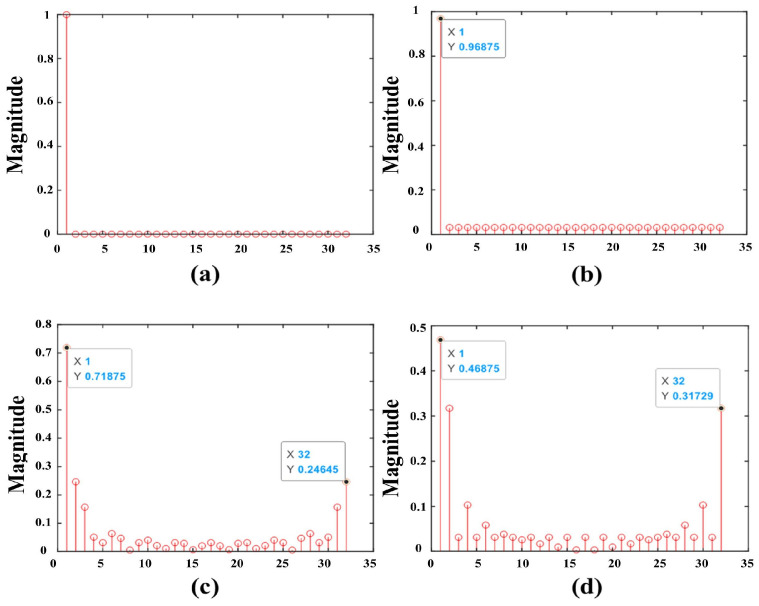
A schematic diagram of the ZC correlation operation for different sequences. (**a**) Two complete ZC sequences are related, (**b**) one of the incomplete ZC sequences is zero, (**c**) one-quarter of the incomplete ZC sequence is zero-valued, and (**d**) half of the incomplete ZC sequence is zero-valued.

As shown in Figure 4a, with $N_{1}=32$, for example, a peak occurs when and only when two complete ZC sequences are aligned, and the correlation value is zero at the rest of the positions. However, if one of the ZC sequences is complete but the other one is incomplete, some secondary peaks interfere. Figure 4b shows the result of an incomplete ZC sequence with one value set to zero $\left(C\left(k\right)=0,k=N_{1}-1\right)$ and shifted with the complete ZC sequence; Figure 4c shows the result of an incomplete ZC sequence with one-quarter of a value set to zero $\left(C\left(k\right)=0,0.75N_{1}\leq k\leq N_{1}-1\right)$ and shifted with the complete ZC sequence; Figure 4d shows the result of an incomplete ZC sequence with a half value set to zero $\left(C\left(k\right)=0,0.5N_{1}\leq k\leq N_{1}-1\right)$ and shifted with the complete ZC sequence. The result of shift correlation shows that the greater the number of zero values in the incomplete ZC sequence within a certain range, the more serious the interference of secondary peaks. If the correlation operation is performed directly on the received signals in the above range without filtering out the noise, it will produce sub-peak interference judgments and affect the accuracy of channel estimation.

The results of Equation (18) are correlated and channel estimation is performed based on the resulting peaks.

(19) $$\hat{h}[l,k]=\{\begin{array}{cc} \;\;\;Q, & |Q|\geq \lambda \;\;\\ 0, & \mathrm{otherwise} \end{array}$$

where $Q=\sum_{n=0}^{N_{1}-1}Y^{\mathrm{DD}}[l,k+n]C^{*}(n)/N_{1},Q\geq |\lambda |$ (λ is the judgment threshold); at this time, the channel gain estimate for path i is ${\hat{h}}_{i}=\hat{h}[l,k]$, the amount of channel delay is ${\hat{l}}_{i}=l-l_{p}$, and the channel Doppler shift is ${\hat{k}}_{i}=k-k_{p}$. Multiple-channel estimation is more flexible than single-pilot channel estimation because the inserted leads are not limited by the peak-to-average ratio, and the number of leads can be increased according to the actual application.

**Figure 5:**
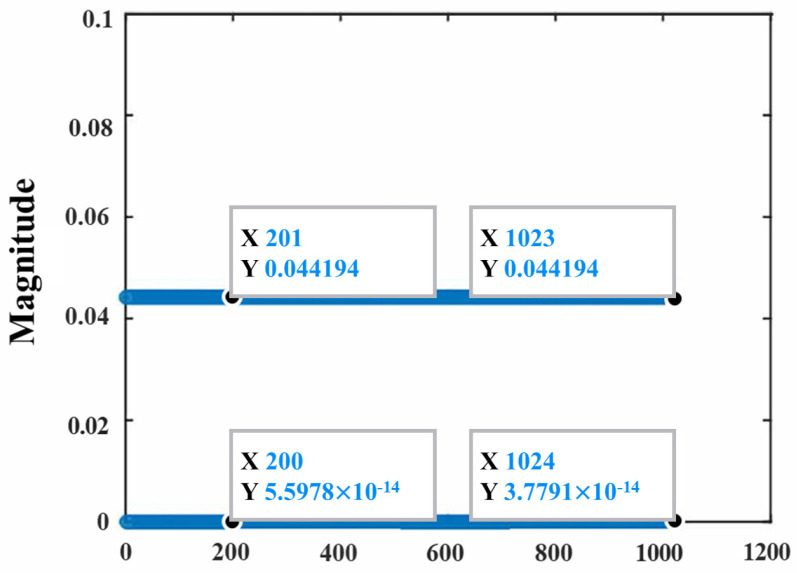
The two ZC sequences r = 1 and r = 3 are correlated with each other.

The multiple-channel estimation scheme based on CAZAC sequences proposed in this paper can be extended to the MIMO-OTFS system, and MIMO-OTFS channel estimation is achieved through the orthogonality of ZC pilot sequences on each antenna. The orthogonality between the antennas is mainly achieved by choosing different r values for the ZC sequences. Figure 5 gives a schematic diagram of two ZC sequences correlated with each other for $N_{1}=1024,$ r=1, and r=3. It can be found that the results of the two sequences correlated with each other are very small, and the two sequences can be approximated as orthogonal at this time. Based on the orthogonality of ZC sequences of different antennas, the total number of guide frequencies and protection intervals required for each antenna is $\begin{array}{c} (2l_{\tau }+1)\left(4k_{v}+N_{1}\right) \end{array}$. Unlike the single-pilot scheme, the protection interval needs to be increased to maintain the orthogonality of the pilots as the number of antennas increases. The proposed multi-pilot channel estimation scheme can effectively improve the spectral efficiency of the system. Due to the limitation of space, MIMO-OTFS multi-pilot channel estimation is not discussed in this paper.

## 4. Experiments

### 4.1. Experimental Details and Metrics

In this section, the proposed signal detection algorithm is simulated numerically, and the main parameters used in the simulation are shown in Table 1. At the receiver side, it is assumed that the channel state information is known, and the channel corresponding to one path for one delay amount is considered in the simulation. The Doppler shift combined with the maximum velocity is randomly generated by the Jakes model, and this paper focuses on the integer Doppler shift case.

In this paper, we select the NMSE as the evaluation metric for the experiments.

(20) $$\mathrm{NMSE}=E\left\{\frac{∥\hat{H}-H{∥}_{2}^{2}}{∥H{∥}_{2}^{2}}\right\}$$

where $\hat{H}\in C^{M\times N}$ denotes the estimate of the DD domain channel matrix $H\in C^{M\times N}$.

### 4.2. Experimental Result and Analysis

**Figure 6:**
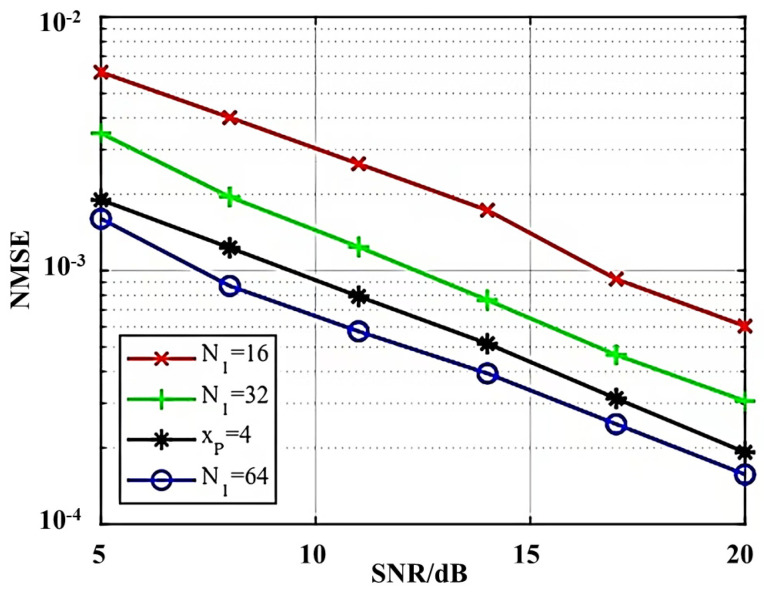
Performance comparison of single-pilot channel estimation and NMSE based on ZC sequence multi-pilot channel estimation.

Figure 6 compares the normalized mean square error (NMSE) of single-carrier channel estimation in the literature [14] and the ZC sequence-based multi-pilot channel estimation proposed in this paper. The pilot energy is equal for the ZC sequence length of 16 $\left(N_{1}=16\right)$ and the single-pilot amplitude of 4, but the average power of the single-pilot case is greater than that of the multi-pilot case, and from the figure, it is found that the performance is better than ZC sequence-based channel estimation. When the length of the ZC sequence is increased to 64, the performance is better than the single-pilot frequency with a frequency amplitude of 4. This is the advantage of ZC sequence multi-pilot frequency channel estimation, as it can improve the performance by increasing the sequence length without the amplitude limitation.

**Figure 7:**
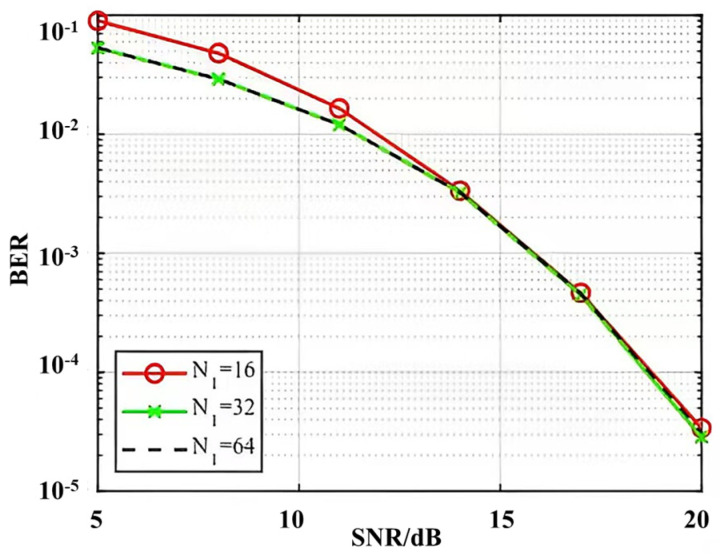
BER performance comparison of ZC sequences of different lengths.

BER performance continues to improve with increasing ZC length, as shown in Figure 6, but the BER curves almost overlap for $N_{1}=32$ and $N_{1}=64$ in Figure 7, and increasing the ZC sequence length does not result in an improvement in BER performance. The three BER curves almost overlap between 16 db and 20 db. The simulation results show that the proposed algorithm can achieve high-accuracy channel estimation, and the channel estimation performance can be improved by increasing the length of the CAZAC sequence.

## 5. Conclusions

This paper proposes a novel channel estimation method for the OTFS system. The main contributions are as follows: The input–output relationship of the OTFS system is derived, and the equivalent channel matrix expression is presented. The time-invariant characteristics of the delay Doppler domain channels are analyzed. To address the issues of missed path detection and excessive fading coefficient estimation errors in single-pilot channel estimation for OTFS systems, a CAZAC sequence-based multi-pilot OTFS channel estimation scheme is proposed.

The proposed method inserts pilots along the Doppler domain and applies denoising techniques at the receiver to enhance the estimation accuracy. The numerical simulation results validate the effectiveness of the proposed algorithm and demonstrate that the channel estimation performance can be further improved by increasing the length of the CAZAC sequences.

The theoretical analysis and simulation results fully verify the superiority of the CAZAC sequence-based multi-pilot OTFS channel estimation method. This technique provides a promising solution that will enable reliable high-speed communications in future mobile networks. Future work may further explore the application of this method in multi-antenna MIMO-OTFS systems. When designing the signal detection algorithm, on the one hand, it is necessary to consider how to reduce the complexity of the detection algorithm in large-scale MIMO-OTFS systems, and on the other hand, it is necessary to consider the signal interference caused by different antennas. When designing the channel estimation scheme, it is necessary to consider additional spatial dimensions and analyze the sparsity of the three dimensions. In addition, since our scheme needs to set the protection interval, the algorithm of the superposition of guide frequency symbols and data symbols can be considered in subsequent research to improve the complexity of the channel estimation algorithm. The data symbol superposition algorithm improves the spectral efficiency, but it has to deal with the interference caused by the data symbols [26].

## Figures and Tables

**Table 1:** Simulation experiment parameters.

Parameters	Parameter Value
Carrier frequency (GHz)	4
Number of carriers (M)	256
Number of symbols (N)	256
Carrier interval (kHz)	15
Maximum speed (km/h)	500
Loop prefix length	17 (6.67%)
Symbol modulation	4-QAM
Number of paths (P)	4

## Data Availability

The original contributions presented in this study are included in the article/Supplementary Materials; further inquiries can be directed to the corresponding authors.

## Supplementary Materials

The following supporting information can be downloaded at: https://www.mdpi.com/article/10.3390/s24237588/s1.
